# Mobilizable antibiotic resistance genes are present in dust microbial communities

**DOI:** 10.1371/journal.ppat.1008211

**Published:** 2020-01-23

**Authors:** Sarah Ben Maamar, Adam J. Glawe, Taylor K. Brown, Nancy Hellgeth, Jinglin Hu, Ji-Ping Wang, Curtis Huttenhower, Erica M. Hartmann

**Affiliations:** 1 Department of Civil and Environmental Engineering, Northwestern University, Evanston, Illinois, United States of America; 2 Department of Statistics, Northwestern University, Evanston, Illinois, United States of America; 3 Department of Biostatistics, Harvard T.H. Chan School of Public Health, Boston, Massachusetts, United States of America; UNITED KINGDOM

## Abstract

The decades-long global trend of urbanization has led to a population that spends increasing amounts of time indoors. Exposure to microbes in buildings, and specifically in dust, is thus also increasing, and has been linked to various health outcomes and to antibiotic resistance genes (ARGs). These are most efficiently screened using DNA sequencing, but this method does not determine which microbes are viable, nor does it reveal whether their ARGs can actually disseminate to other microbes. We have thus performed the first study to: 1) examine the potential for ARG dissemination in indoor dust microbial communities, and 2) validate the presence of detected mobile ARGs in viable dust bacteria. Specifically, we integrated 166 dust metagenomes from 43 different buildings. Sequences were assembled, annotated, and screened for potential integrons, transposons, plasmids, and associated ARGs. The same dust samples were further investigated using cultivation and isolate genome and plasmid sequencing. Potential ARGs were detected in dust isolate genomes, and we confirmed their placement on mobile genetic elements using long-read sequencing. We found 183 ARGs, of which 52 were potentially mobile (associated with a putative plasmid, transposon or integron). One dust isolate related to *Staphylococcus equorum* proved to contain a plasmid carrying an ARG that was detected metagenomically and confirmed through whole genome and plasmid sequencing. This study thus highlights the power of combining cultivation with metagenomics to assess the risk of potentially mobile ARGs for public health.

## Author summary

Controlling the spread of antibiotic resistance is a complex, high-priority global challenge. Antibiotic resistance is difficult to control because it is present in so many environments and because antibiotic resistance genes can transfer between different bacteria. The built environment is important to consider because that is where people in developed countries spend the majority of their lives. Previous studies have found antibiotic-resistance genes in indoor microbiomes, generating concern that the indoor microbiome could serve as a reservoir for transferrable antibiotic resistance genes. Using metagenomic sequencing, we identified potentially mobile antibiotic resistance genes in indoor dust microbial communities. We further identified these same genes in bacteria isolated from the same indoor dust and demonstrated that they are located on mobile genetic elements. While our results support the hypothesis that the indoor microbiome harbors mobile antibiotic resistance genes, we note that the relative abundance of antibiotic resistance genes in indoor dust is quite low compared to other environments.

## Introduction

Antibiotic resistance is a public health priority worldwide, to the point where the World Health Organization has forecasted an entry into a post-antibiotic era within the current century [1]. Antibiotics are intensively used from agriculture to medicine and have become widespread in the environment, enriching resistance factors in areas as diverse as hospitals, cattle stool, wastewater treatment plants, and drinking water [2–4]. In some settings, such as healthcare environments, the presence of antimicrobial resistant organisms is a direct risk to human health, either due to frank pathogens or organisms that become opportunistic in immunocompromised patients [5, 6]. Much more prevalently, however, antimicrobial resistance elements can remain resident in otherwise harmless organisms in everyday indoor environments [7–10]. It thus becomes difficult to determine the latent health risk of microbes or individually mobile resistance elements with the potential to induce resistant infection later or in other hosts.

While the spread of antibiotic resistance is primarily associated with hospitals, antibiotic resistance genes have been detected in soil, freshwater, sediments, cattle stool, human gut, wastewater treatment plants, and hospitals [2, 4, 11–15]. Genes related to antibiotic resistance were also more recently detected in outdoor [16, 17] and indoor dust [8, 18, 19]. ARGs in built environments are of particular relevance since humans in urban areas spend ~90% of their lifetime indoors [20], and by 2050 66% of the world population is predicted to live in cities (https://www.urbanet.info/world-urban-population/). Studies of microbial diversity within buildings have primarily focused on hospital settings [21–24] to assess e.g. the prevalence of pathogens. Indoor microbial diversity has also been characterized in other confined environments, such as the International Space Station [25–27], but without a focus on mobile ARG in these environments. The few such studies have found ARGs with comparable diversities to hospitals or agricultural settings, including *tet*(W), *blaSRT-1*, *erm*(B), *qnr*, *ftsH* and *mtrAB* encoding respectively resistance to tetracycline, beta-lactams, macrolides, fluoroquinolones, aminoglycosides, and multidrug resistance (efflux pumps) [8, 9]. While the effects related to the presence of these ARGs have been closely studied in hospitals [28–34], little information is available regarding the prevalence or transmissibility of ARGs in public spaces and individual households.

Such studies are further complicated by the fact that many ARGs are intrinsic to bacterial genomes, particularly in the environment; they are part of the so called “intrinsic resistome” and were not acquired in response to anthropogenic antimicrobial exposures [35]. Correspondingly, such ARGs do not necessarily always confer antibiotic resistance [36–37]. Because “intrinsic ARGs” are typically located on the bacterial chromosome, and not *a priori* associated with any mobile genetic element, they remain confined to a specific taxon. Such genes can be a threat for public health if they are expressed; however, the impact of these genes can be directly limited by controlling exposures to the specific organisms carrying the gene. However, under environmental pressures, some intrinsic ARGs might be mobilized via integration in a mobile genetic element (e.g., transposase, integrase or plasmid) and transferred to other bacteria. Mobilized ARGs are commonly referred to as “acquired” [36] and have been observed in the dissemination of antibiotic resistance in clinical settings and other highly selective environments. One study, for example, demonstrated that plasmids carrying various ARGs, such as tetracycline, trimethoprim, and kanamycin resistance determinants, could invade a highly diverse fraction of soil bacteria with little host specificity [38, 39]. Therefore, to fully understand and attempt to predict dissemination of ARGs, it is necessary to characterize individual strains of bacteria, including chromosomal and mobile elements, and to evaluate the associated resistance phenotypes functionally whenever possible [40]. Given the widespread presence of antimicrobials in dust, it is possible that this environment selects for and enhances the transfer of mobile ARG [8, 9]. However, it is unknown whether the ARG detected in dust are in fact mobile and if they are even present in viable bacteria.

We thus present here a joint metagenomic and cultivation study of mobilizable ARGs from built environment microbial communities. We characterized the mobile resistomes of 166 dust metagenomes from samples spanning 43 different buildings. This allowed us to assess spatial associations in metagenomic contigs between specific mobile elements and ARGs, inferring which ARGs had either been mobilized or were potentially mobilizable. These were investigated in parallel using bacterial isolates cultured from the same dust samples. After phenotypic characterization and identification via short-read and long-read sequencing of isolates carrying potentially mobile ARGs, this identified an unexpectedly chromosomal element (*gidB*) and a plasmidic resistance element (*lnuA*) that putatively encode streptomycin and lincosamide resistance, respectively. Taken together, the joint culture-independent and -dependent approaches identify a pool of potentially mobilizable and functional antimicrobial resistance elements in indoor dust microbial communities.

## Materials and methods

### Metagenome analysis and screening for mobile genetic elements and associated ARGs

A total of 166 dust metagenomes were analyzed in this study, including 122 metagenomes published in Fahimipour et al., 2018 [9] and 44 samples from Hartmann et al., 2016 [8] (in the Sequence Read Archive under accession no. PRJNA489265 and PRJNA321035, respectively). In addition to these primary discovery datasets, metagenomes from Ma et al., 2017 [4] and from Hu et al., 2016 [2] were additionally analyzed for later comparisons.

The metagenomic raw reads from all datasets were trimmed and quality-controlled with Kneaddata v. 0.5.1 (http://huttenhower.sph.harvard.edu/kneaddata) with default parameters. Quality controlled-reads from all datasets were processed with ShortBRED v. 0.9.5 (default parameters) [41] using the Comprehensive Antibiotic Resistance Database v.1.05 [42] as a reference database to define the composition and abundance in antibiotic resistance genes of each sample from each dataset. Quality controlled-reads of dust, drinking water, and cattle stool datasets were used to run HUMAnN2 v. 0.11.1 [43] to determine transposase, integrase, and plasmid-related gene abundances. These were identified as any UniRef90 [44] including the strings “integrase,” “integron,” or words beginning with “transpos” or “plasmid.”

Dust dataset quality-controlled reads were assembled using the Kiki assembler v. 0.0.12 (default parameters, https://github.com/GeneAssembly/kiki) in Kbase [45]. Contigs longer than 3 kb were retained and annotated with Rast (default parameters) [46], and potentially mobile genetic elements (MGE) present in the annotated contigs of each dust metagenome were annotated as above. The coverage of each mobilizing enzyme (i.e. transposase, integrase) gene or each plasmid-related gene was quantified by mapping reads against the portion of the contig coding the mobilizing protein or the plasmid-related gene using FR-HIT v. 0.7.1 [47] and summarized using the Bedtools suite tools v. 2.27.1 [48]. The length-normalized average depth of coverage of each mobilizing enzyme gene or plasmid related gene was then used as a proxy for its abundance [49].

To assess the effect of sequencing depth and assembly on the number of MGE detected in dust datasets, the average coverage in each sample was computed using Nonpareil with default parameters and with the following options -T kmer -f fastq [50]. The N50 value and total contigs count were recovered from the Kiki assembler output for each dust sample. Pearson correlation between the total MGE count detected in each dust sample with the average coverage, the N50 value, and the total number of contigs was calculated using R ggpubr and vegan R packages.

To link MGEs and ARGs, each contig containing an MGE was manually examined and checked for the presence of potentially mobile ARGs nearby (5 kb before and after the mobilizing enzyme gene or the plasmid-related gene) based on the presence of an ARG from the CARD database, in order to avoid false positives. The position of each potentially mobile ARG was recorded. Abundance of each putative mobile ARG was assessed as above. The aggregate abundance of all MGEs in each respective dataset was calculated as the sum of all transposases, integrases, and plasmid-related genes detected in all the samples of each respective dataset (dust, drinking water, and cattle stool). The same calculation was used to determine the total abundance of ARGs in each dataset.

### Short-read, long-read, and 16S rRNA gene sequencing of isolates

The sample in which metagenomic data indicated the presence of a plasmid and a high proportion of *Staphylococcus* genus-associated reads (~55%) was selected for further screening to identify potential members of the *Staphylococcus* genus. Isolates from this sample (see Fahimipour et al., 2018 [9] for full details) were screened based on their carriage of coccoidal morphology (grape-like clusters of cocci with usually yellowish colonies) with optical microscopy and positive Gram staining. Of 19 total isolates cultured from the sample, a single isolate showed *Staphylococcus*-type morphology by these criteria.

DNA was thus extracted from this sample (ID “AF82B3”) using the DNeasy PowerSoil Kit from Qiagen following the manufacturer’s instructions, for Sanger sequencing from the forward end of the 16S rRNA gene amplified with the 27f primer. The resulting 16S rRNA sequence was blasted (blastn) against the nr NCBI database to assign taxonomy to the identified strain, which was most closely related to *Staphylococcus equorum* (100% full-length 16S rRNA gene identity). Genomic DNA libraries were prepared using the Illumina Nextera XT and Index Kits following the manufacturer’s protocol. The whole genome sequence of the isolate was sequenced on an Illumina MiSeq (2x150 bp) using extracted total genomic DNA. The reads were trimmed and quality-controlled with Kneaddata (http://huttenhower.sph.harvard.edu/kneaddata) as described above and assembled using SPades assembler v. 3.11.1 [51] with default parameters to reconstruct a full genome. Taxonomy of the genome was assigned using SpeciesTreeBuilder v. 0.0.12 [52] that compares the genome with different reference publicly available bacterial genomes from NCBI. The genome was also assigned to *Staphylococcus equorum* with this strategy.

In order to determine the presence of plasmids and their sequences in this specific dust-borne *S*. *equorum*, total plasmid DNA was extracted from 120 mL of culture grown in TSB medium at 37°C for 24–48 hours using QIAprep Spin Miniprep Kit (Qiagen, Germantown, MD). The extracted plasmid DNA was then quality controlled and quantified using the Quant-iT PicoGreen dsDNA Assay Kit (Invitrogen, Carlsbad, CA) and processed to construct Nanopore long-read library with the Nanopore Rapid Barcoding Kit using 310 ng of extracted plasmid DNA. The plasmid DNA libraries were sequenced on Oxford Nanopore MinION and a total of 20,365 long-read sequences were produced with an average length of 7,000 bp. The long reads were corrected, trimmed and assembled using Canu v. 1.3 [53] with the following specific parameters to optimize plasmid assembly: corMinCoverage = 0 and corOutCoverage = 1000. The unitigs generated by Canu were further polished using Pilon v. 1.20 [54]. This process resulted in 6 long contigs (called “unitig”): unitig 3 (77,695 bp); unitig 5 (53,438 bp); unitig 7 (17,250 bp); unitig 10 (3,686 bp); unitig 18,655 (12,590 bp); and unitig 18,656 (2,669,698 bp).

### Minimum inhibitory concentration (MIC) determination of *Staphylococcus equorum* dust isolate and *Staphylococcus aureus* ATCC 25923

Growth curves in Tryptone Soy Broth at 37°C for the *S*. *equorum* dust isolate used as plasmid donor and *S*. *aureus* ATCC 25923 lab strain used as plasmid recipient in the transferability assays (conjugation or transfer) were performed over 24 hours during which the optical density at 600 nm was measured every hour. These tests allowed the determination of each strain’s midpoint of exponential growth phase, which was at 24 h and 30 h for S. *equorum* and *S*. *aureus* ATCC 25923, respectively.

### Minimum inhibitory concentration (MIC) and Kirby Bauer susceptibility determination of *Staphylococcus equorum* dust isolate and *Staphylococcus aureus* ATCC 29213 (http://www.eucast.org/clinical_breakpoints/)

For MIC measurements, a culture of 5 mL was started in cation-adjusted Mueller Hinton broth for the *S*. *equorum* dust isolate or *S*. *aureus* ATCC 29213 from 3 colonies previously grown on a cation-adjusted Mueller Hinton plate at 37°C. The tested antibiotics include clindamycin, streptomycin, ampicillin, chloramphenicol, kanamycin, tetracycline and ciprofloxacin. All antibiotics except chloramphenicol were tested at the following concentration range: 0, 0.015625, 0.03125, 0.0625, 0.125, 0.25, 0.5, 1, 2 mg/L. Chloramphenicol was tested at concentrations up to 64 mg/L. An inoculum concentration of approximately 5x10^5^ CFU/mL was used for both *S*. *equorum* and *S. aureus* ATCC 29213. Growth was monitored through measurement of optical density at 600 nm after 20 hours incubation at 37°C. MIC results were determined based on clinical breakpoints documented by European Committee on Antimicrobial Susceptibility Testing [55] (*http://www.eucast.org/clinical_breakpoints/*).

For Kirby Bauer susceptibility testing, *S*. *equorum* and *S*. *aureus* ATCC 29213 were grown in cation-adjusted Mueller Hinton broth to an optical density at 600 nm equivalent to McFarland 0.5 turbidity standard. A sterile swab was dipped in an inoculum tube and streaked three times over the entire plate surface, as described in (https://www.asmscience.org/content/education/protocol/protocol.3189). Antibiotic disks were placed on the center of the agar surface, using flamed forceps. All plates were incubated for 20 hours at 37°C.

Complete results of the bioinformatic analyses are available at https://figshare.com/projects/Mobilizable_antibiotic_resistance_genes_are_present_in_dust_microbial_communities/70184.

## Results

### Potentially mobile ARGs are present in dust microbial communities

**Fig 1:**
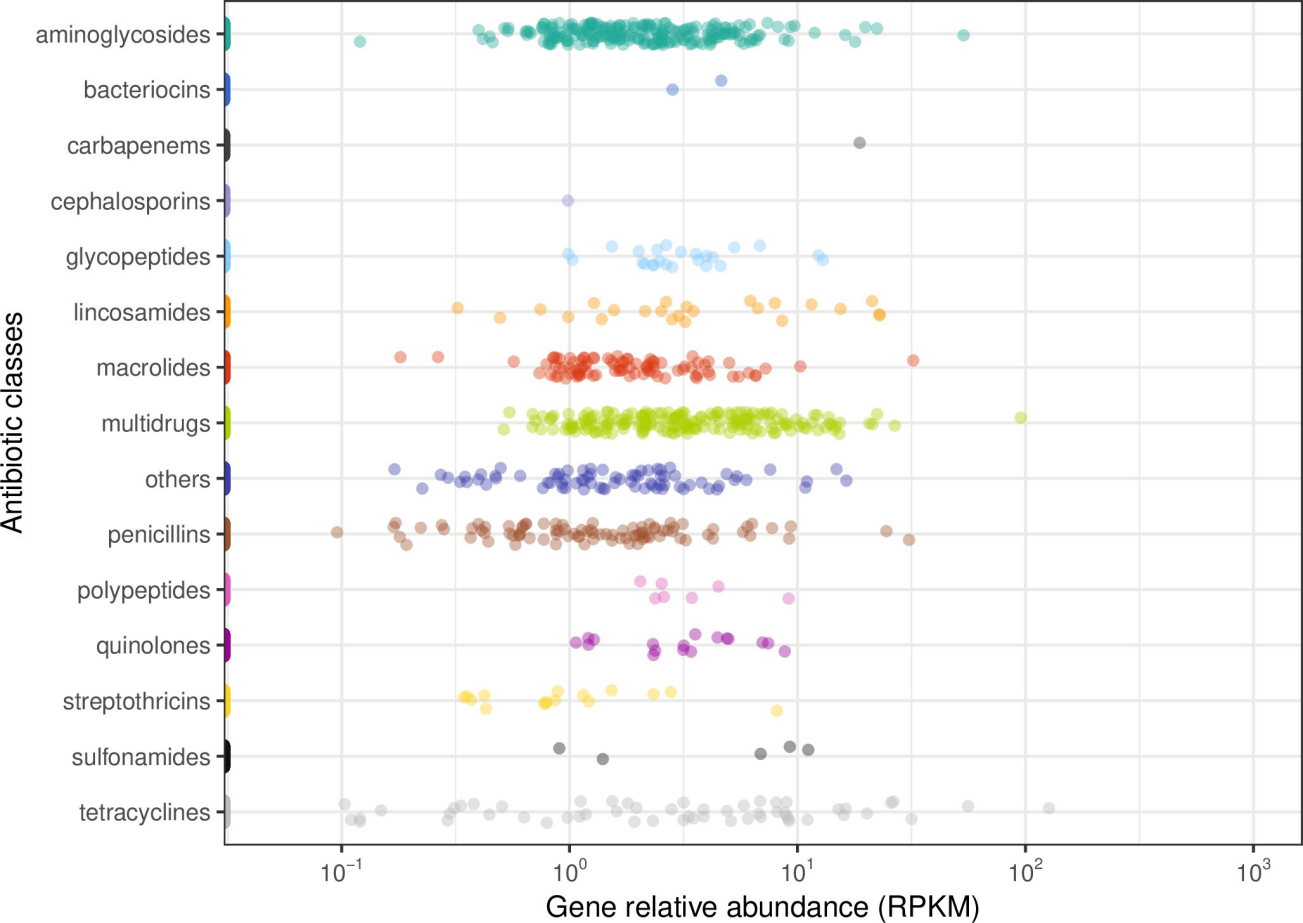
Antibiotic resistance gene (ARG) class relative abundances in dust metagenomes. ARGs were quantified using ShortBRED [41] from 166 total dust metagenomes spanning 43 buildings [8, 9]. Here, the relative abundance of individual ARGs over all dust samples (normalized as reads per kilobase per million mapped reads (RPKM) are grouped by class according to CARD [42], each circle indicating the relative abundance of one single ARG in a single sample belonging to a specific antibiotic class. The colors indicate the different antibiotic classes.

We jointly analyzed two dust metagenome collections using a uniform assembly process, one [8] containing 44 samples spanning various sites within a single athletic facility, and the second [9] including 122 samples from numerous sites at 42 different facilities (**Fig 1**). The dust metagenome collection sites were described in Hartmann et al., 2016 [8] and Fahimipour et al., 2018 [9]. Each sample in both datasets was independently quality controlled by KneadData (http://huttenhower.sph.harvard.edu/kneaddata), assembled using Kiki (https://github.com/GeneAssembly/kiki), and ARGs identified and quantified using ShortBRED [41] with CARD [42] as a reference (see **Methods**).

**Fig 2:**
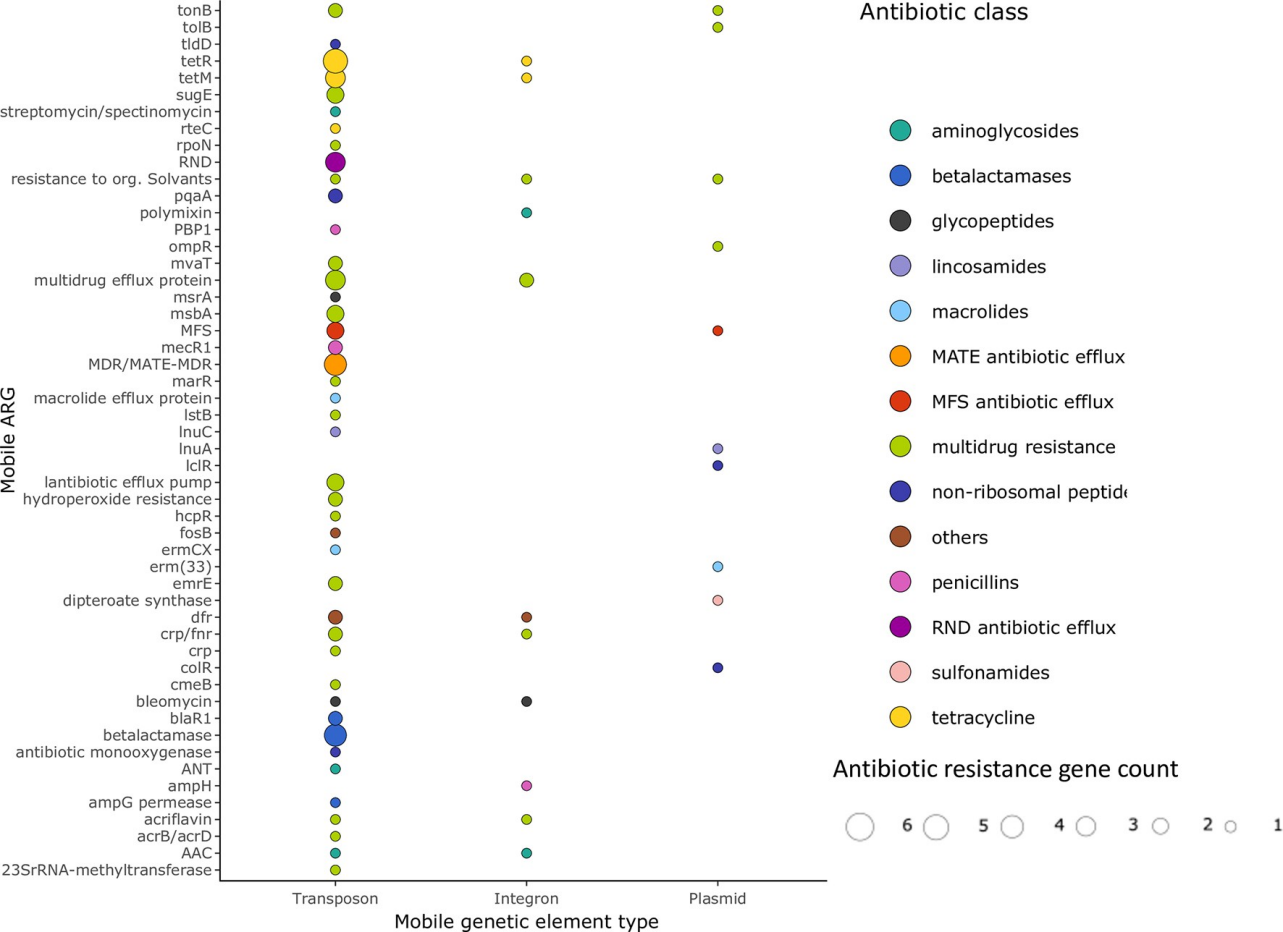
Most individual ARGs are rarely mobilizable in dust metagenomes. ARGs were rarely detected near potentially mobilizable elements in dust metagenome assemblies, and were not significantly differentially distributed across mobile element types (since transposons were also most commonly detected independently of ARGs, see text). Point size indicates the total occurrences of each ARG type per mobilization mechanism across all samples.

In the corresponding joint set of 166 dust metagenomes, a total of 183 different ARGs were detected. These represent resistance to 15 different antibiotic classes, including multidrug resistance, aminoglycosides, tetracyclines, macrolides, penicillins, cephalosporins, and others (**Fig 1**, **Table 1**). We additionally assessed whether these ARGs were potentially mobile (or mobilizable) in each sample’s corresponding metagenomically assembled contigs (see **Methods**) by flagging all open reading frame annotations indicative of potential transposable elements,” integrons, or plasmids and identifying ARGs within 5 Knt (**Fig 2**).

**Table 1:** Frequent ARGs in dust microbial communities. Abundances 20 most frequently detected ARGs from ShortBRED [41] are reported, excluding samples were the gene was not detected; prevalence is indicated as the percentage of the 166 total dust samples in which the gene was detected.

Top 20 ARG	Antibiotic class	Median abundance (RPKM)	Percentage of samples
AAC(3)-VIIa	aminoglycosides	2.41	44
ErmO	macrolides	1.20	24
CRP	**multidrugs**	5.00	23
AAC(3)-VIIIa	aminoglycosides	3.27	17
qacA	**multidrugs**	2.92	14
APH(3_)-Ia	aminoglycosides	2.05	14
vanRO	glycopeptides	2.74	11
tetO	tetracyclines	6.80	10
PC1_beta-lactamase_(blaZ)	penicillins	2.08	10
lnuA	lincosamides	3.14	6
dfrC	others	5.30	5
emrR	**multidrugs**	5.63	5
tetW	tetracyclines	15.95	3
mexT	**multidrugs**	14.73	3
acrD	aminoglycosides	17.89	2
CfxA6	penicillins	14.40	2
acrB	**multidrugs**	12.75	2
tet32	tetracyclines	4.43	2
cpxA	**multidrugs**	10.95	2
tetQ	tetracyclines	76.58	1

Of the 2,614 total contigs containing potentially mobile genetic elements (MGE), the largest proportion were putative transposable elements (77%), followed by integrons (16%) and plasmids (7%; **S1 Fig**). Eighty-seven of these contigs containing MGEs also contained ARGs, of which most were multidrug resistance genes (64%) and antibiotic efflux pumps of the Resistance-Nodulation-Division (RND; 5%), multi-antimicrobial extrusion (MATE; 6%) and major facilitator superfamily (MFS; 6%) types; these genes were found in all three types of MGE. In total, there were 57 different potentially mobile ARGs detected, of which 18 were related to multidrug resistance (including efflux pumps) and 83% were found in multiple (>1) dust samples. The gene *CRP*/*Fnr*, which encodes a multidrug efflux pump, and other genes coding for multidrug efflux protein, were the most frequently detected mobile multidrug resistance gene (detected in four samples). These genes are also among the most frequent ARGs (e.g. *crp*) over all dust samples (**Table 1**). We estimated that samples were sequenced at approximately 30% saturation using Nonpareil [50], meaning that these values represent minima and are likely underestimates of true ARG diversity in dust (**S2 Fig**). The effect of coverage on our analysis was significant but minor: we detected more MGE in samples with higher coverage (*p*-value = 0.023; r = 0.18).

Of the top 20 most frequent resistance genes (**Table 1**), 6 were associated with multidrug resistance. Two of the six multidrug resistance genes, *crp* and *qacA*, were relatively abundant and particularly prevalent in dust samples. These genes are related to multidrug efflux: the *crp* gene encodes a global regulator repressing expression of the *mdtEF* RND multidrug efflux pump gene [56, 57], while the *qacA* gene encodes a subunit of the Qac MFS type multidrug efflux pump [58]. Those two genes are particularly well represented in dust microbial communities exposed to antimicrobials, and could in principle play a role in resistance or persistence in dust. CRP is typically associated with *Escherichia coli* [56, 57], and QacA is typically associated with *Staphylococcus* [58]. Using all against all association testing, we identified significant associations between these genes and their typical hosts (**S3 Fig**). The MATE multidrug efflux pump gene was the only potentially mobile multidrug resistance gene found exclusively in transposons.

The next most common classes in the 20 most abundant ARGs were genes related to aminoglycoside resistance and tetracycline, with four genes each. Although aminoglycoside resistance genes were most frequent across all dust samples (e.g. *AAC(3)-VIIa*; *AAC(3)-VIIIa* and *APH(3)-Ia*) (**Table 1**), these genes were not frequently detected in the mobile ARGs (**Fig 2**). The tetracycline resistance genes *tetQ* and *tetW* (mobile or not mobile) were highly abundant, although they were only detected in a few samples. These genes were not detected in association with MGE, but other genes related to tetracycline resistance (*tetM*, *tetR*) were (**Fig 2**). *tetW* was only associated with two taxa (*Dorea* and *Parabacteroides*, **S2 Fig**), both of which are human commensals not typical of dust communities (but potentially abundant when deposited in a particular environmental location).

### Dust microbial communities harbor lower abundances of ARGs and potentially mobilizable ARGs compared to other environments

**Fig 3:**
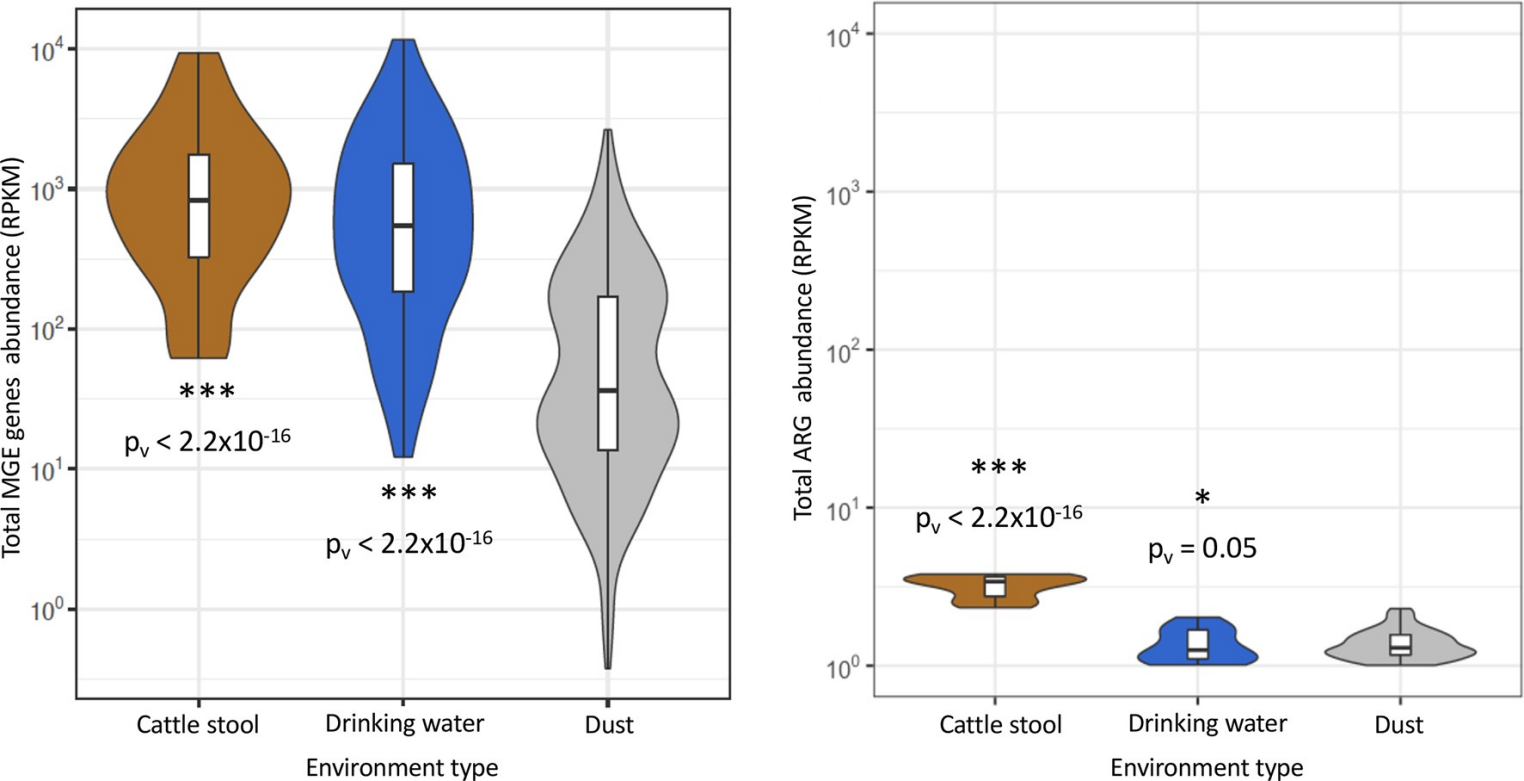
Dust contains few ARGs and mobilizable elements relative to animal gut and drinking water metagenomes. We compared total MGE abundances (sum of all mobilizable gene families from HUMAnN2 [43] profiles and total ARG abundances (sums from ShortBRED [41] profiles) between dust metagenomes and two other datasets: 13 livestock stool samples from Hu et al., 2016 [2] and 25 drinking water metagenomes from Ma et al., 2018 [4]. Animal guts and drinking water had significantly more total MGEs than dust, and animal stool had (as expected) significantly more total ARGs. P-values are shown from Mann-Whitney tests relative to dust distribution.

Total MGE abundances in dust were further calculated using HUMAnN2 [43] (that is, the sum of abundance of all potential mobilization genes; see **Methods**), and compared to total MGEs abundance in drinking water [4] and livestock stool [2]. The same comparison was made using the total ARG abundances (from ShortBRED [41]) in the same three datasets. The total abundance of MGE in dust was significantly (Mann-Whitney p-value < 2.2x10^-16^) lower than in drinking water or in livestock stool. Total abundance of dust ARGs was comparable to that in drinking water (Mann-Whitney p = 0.05), with both significantly (p<2.2x10^-16^) less than that of animal guts (**Fig 3**).

**Fig 4:**
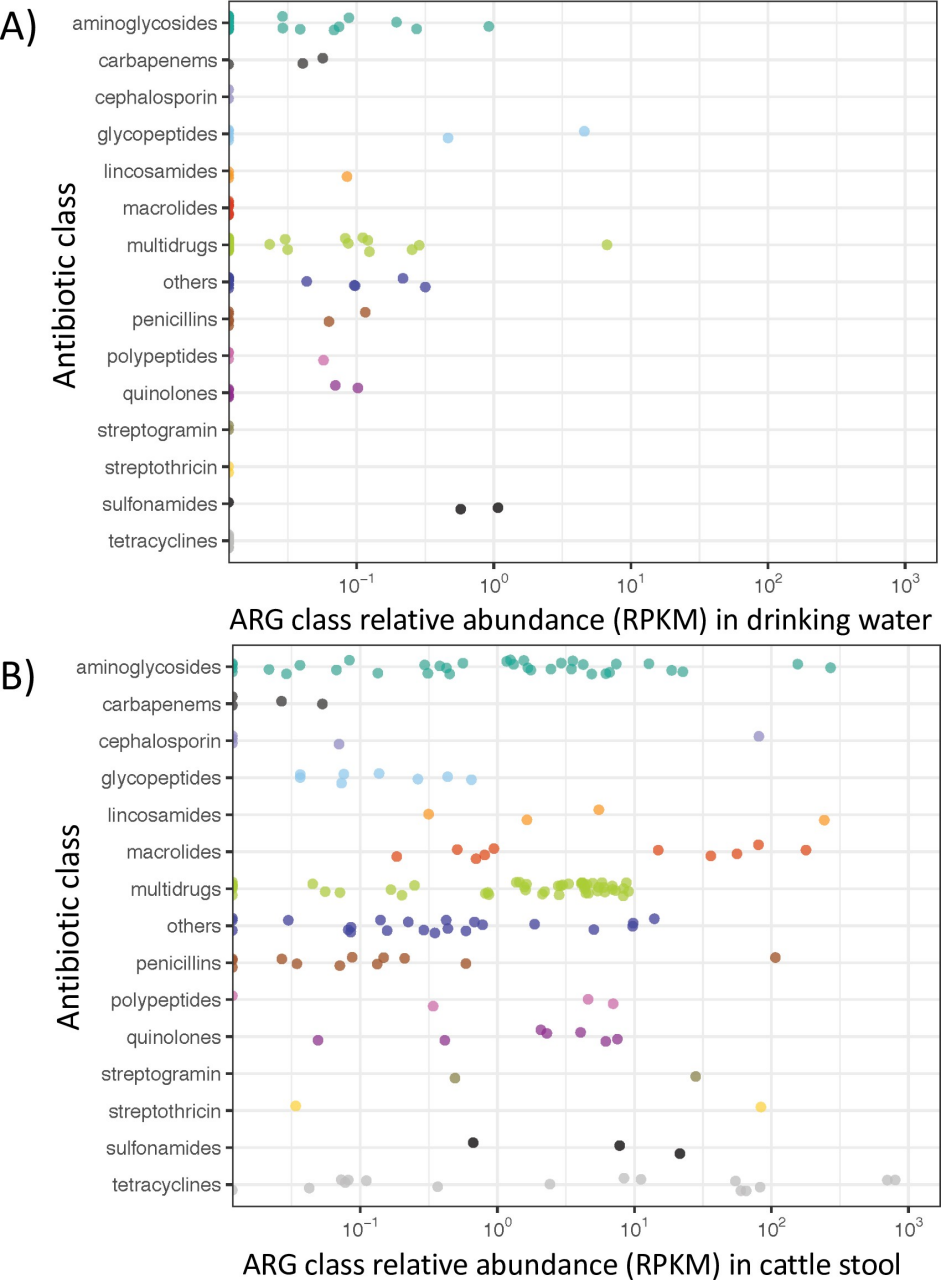
ARG class relative abundances in drinking water and livestock stool metagenomes. ARGs were quantified as for dust in **Fig 1** (average per gene across samples) from 25 drinking water and 13 animal stool metagenomes [2, 4]. ARG levels per class in **A)** drinking water were similar to those in dust, as were overall ARG levels (**Fig 4**), while **B)** in livestock most classes and particularly tetracycline, streptothricin, macrolide, and lincosamide resistance were much more abundant.

**Fig 5:**
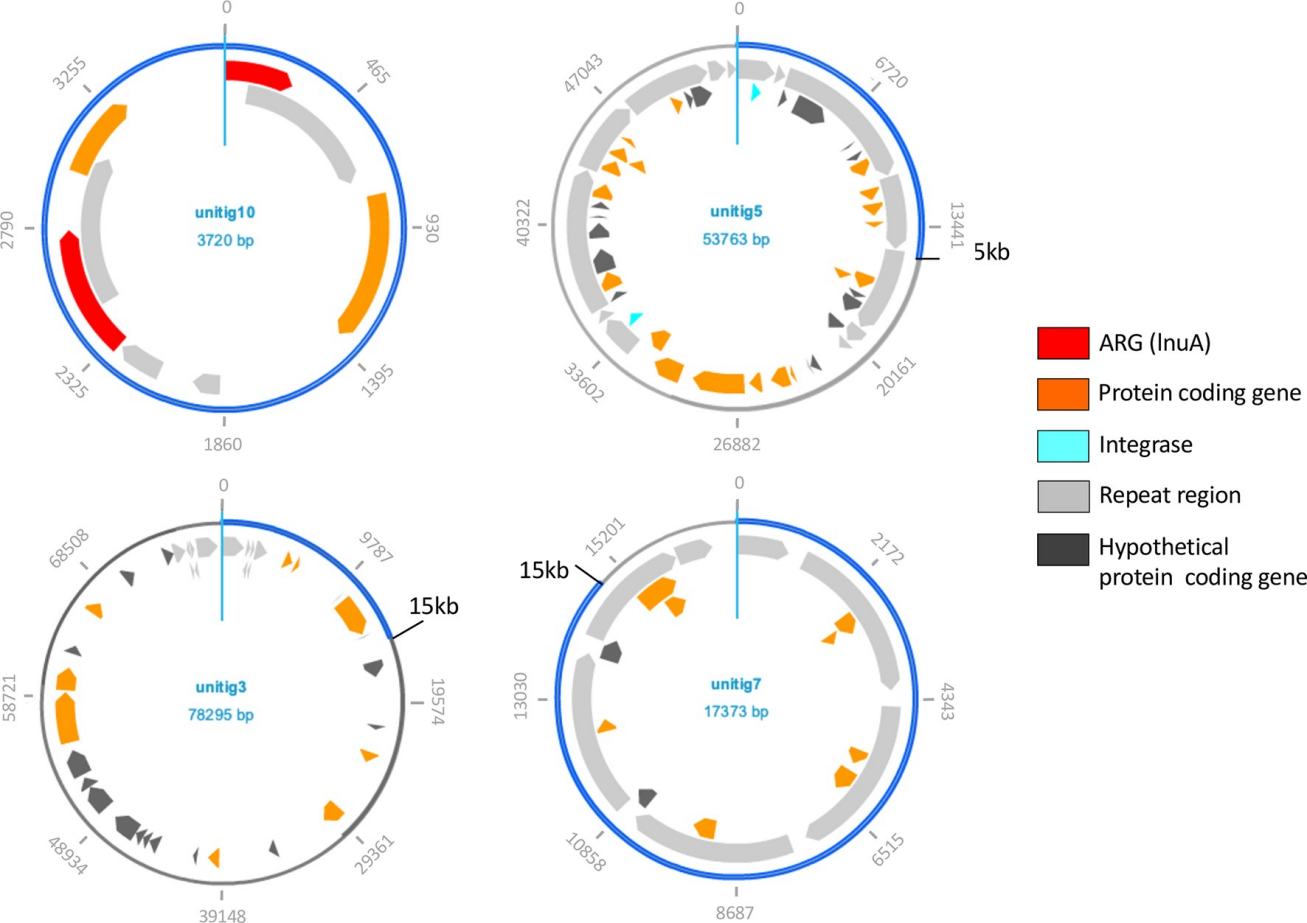
Plasmids reconstructed from long-read sequencing of a cultured dust isolate related to *S*. *equorum*. We targeted staphylococcoidal culture isolates from a dust sample (AF82) with metagenomically abundant *Staphylococcus* to characterize chromosomal and plasmidic ARGs. We identified and sequenced one such strain that proved to be *Staphylococcus equorum* (100% full-length rRNA gene identity). We further isolated its plasmid DNA for Nanopore sequencing (**Methods**), resulting in 4 complete or partial plasmids assembled using CANU / Pilon [53, 54].

We used ShortBRED [41] CARD [42] gene abundance profiles to compare individual ARG class abundances from dust (**Fig 1**) with drinking water and livestock metagenomes (**Fig 4**). Like total ARG abundance, all ARG classes were rare in drinking water (**Fig 5A**), with only sulfonamide resistance occurring at nontrivial levels; other classes, particularly glycopeptide resistance, were comparable to their dust abundances. Most ARG classes were tens to hundreds of times more abundant in animal guts than in dust, as expected, and the distribution of resistance classes shifted to resemble that of the mammalian gut resistome rather than the environmental, non-host-associated microbial profile of dust and drinking water [7].

### Potentially mobile ARG were confirmed on plasmids using culturing and long-read DNA sequencing

Based on its relevance to human health as a commonly antimicrobial-resistant nosocomial pathogen [59], we targeted *Staphylococcus* spp. during culture-based analysis of these dust microbial communities. We targeted a single sample for screening with high abundance (53.59%) of *Staphylococcus* based on the metagenomics and identified isolates with staphylococcoidal morphology (Fahimipour et al. 2019, **Methods** [9]). From that sample, 1 of 19 total colonies had the appropriate morphology and was selected for whole genome sequencing using short-read sequencing and plasmid isolation via long-read sequencing (BioProject ID PRJNA554361). This isolate was classified as *S*. *equorum* based on a BLAST search of the 16S rRNA gene sequence (100% full-length identity).

A total of 4 plasmids were reconstructed from Nanopore sequencing of this *S*. *equorum* dust isolate (**Fig 5**, see **Methods**). When blasted against the NCBI nr database, unitig3 was found the closest to *Staphylococcus kloosii* strain ATCC 43959 plasmid unnamed (E-value = 4.10^−53^; 74.14% identical); unitig5 closest to *Staphylococcus equorum* strain C2014 plasmid C2014-2 (E-value = 0, 93.63% identical); unitig7 to *Staphylococcus saprophyticus* subsp. *saprophyticus* ATCC 15305 plasmid unnamed2 (E-value = 0; 98.94% identical); and unitig10 closest to *Staphylococcus equorum* strain KM1031 plasmid unnamed3 (E-value = 0, 98.77% identical). Sequences annotated as hypothetical proteins were unevenly distributed across plasmids, with the majority being found on unitigs 3 and 5 (14 of 23 total genes in unitig 3, and 15 of 34 total genes in unitig 5, p = 0.039 by Fisher’s exact test). Conversely, unitig 7 contained only 8 detected protein coding genes and 7 repeat regions. Plasmid unitig 5 had the highest number of repeat regions (14) and was the only plasmid on which integrases were annotated.

The smallest plasmid (**Fig 5**) unitig 10 was the sole plasmid on which ARGs were detected, specifically two (of 4 genes in total) that both encode putative lincosamide nucleotidyltransferase (*lnuA*; also called *linA*) genes for resistance to lincosamide antibiotics (of lengths 267 and 486 nucleotides, respectively). These two genes were 98.83% nucleotide identical between each other over 171 nt with 2 gaps. The plasmid itself has a 525 nt repeated region that encompasses parts of both *lnuA* genes and accounts for this 171 nt sequence (**Fig 5**). Only one contig assembled from the corresponding metagenome contained an annotated *lnuA* and was identified as plasmidic. This entire contig (858 nt) aligned with 100% nucleotide identity and 0 gaps to the plasmid sequence obtained from the *S*. *equorum* isolate, although the orientations were reversed. The 486-nt *lnuA* from the plasmid (and accordingly the identical sequence from the metagenome-assembled contig) matched multiple *Staphylococcus*-associated *lnuA* genes in the nr database with 100% coverage and 100% nucleotide identity (e.g., NCBI Reference Sequence NG_050416.1, GenBank J03947.1), as well as identical sequences located on *Staphylococcus*-associated plasmids, many of which have annotated *lnuA* genes.

Based on the assembled contigs, we expected to find the streptomycin resistance gene *gidB* on a plasmid in this organism, as this gene was detected on multiple contigs predicted to originate from plasmids. However, this gene was not detected on any of the assembled unitigs. This strain does indeed carry a *gidB* gene, but it is located on the chromosome. MIC testing further suggests that this gene may not in fact be expressed or confer streptomycin resistance (**Table 2**).

**Table 2:** Summary of the results of the MIC results for the *Staphylococcus equorum* dust isolate. Tests were performed in cation-adjusted Mueller Hinton II broth with *Staphylococcus aureus* ATCC 29213 strain for comparison.

Antibiotic	MIC (mg/L)									
	EUCAST Breakpoint		*Staphylococcus aureus ATCC 29213*				*Staphylococcus equorum*			
	S≤	R>								
Ampicillin*	NA	NA	0.25	0.25	0.25	NA	0.125	0.125	0.125	NA
Chloramphenicol	8	8	8	8	8	S	8	8	8	S
Clindamycin	0.25	0.5	0.002	0.002	0.002	S	0.5	0.5	0.5	S
Ciprofloxacin	1	1	0.125	0.125	0.125	S	0.25	0.25	0.25	S
Kanamycin	8	8	0.0625	0.5	0.0625	S	0.0625	0.125	0.0625	S
Streptomycin*	NA	NA	2	2	2	NA	2	2	2	NA
Tetracycline	1	2	0.016	0.008	0.016	S	0.5	0.5	1	S

* Breakpoints for ampicillin and streptomycin are not documented in the EUCAST Clinical Breakpoint Tables (version 9.0).

Unitig 5 contained two copies each of three genes related to metal resistance: *merR*, *czcD*, and *ACR3*. Two ORFs on unitig 5 were annotated as *merR* sequences, one 299 nt and the other 347. Sequences for the same gene in other organisms are slightly longer (366 nt in *Acidithiobacillus ferrooxidans* (UniProtKB—P22896) and 432 in *Pseudomonas aeruginosa* (UniProtKB—P0A183)). Two ORFs were annotated as *czcD*, both 936 nt, which is longer than the 873-nt reference in Uniprot (UniProtKB—Q1JHF9). Two additional ORFs were annotated as ACR3, one 728 nt and the other 827 nt, both much shorter than homologs found in other species (>1000 nt, e.g., in *Corynebacterium glutamicum* (UniProtKB—Q8NQC8)). MerR transcriptional regulators are classically associated with metal resistance, specifically mercury, but have more recently been linked to regulatory responses to a variety of stresses, including antibiotics [60, 61]. However, the rest of the *mer* operon was not found on this plasmid. CzcD is a regulator for heavy metal ion transport, specifically cobalt, zinc, and cadmium [62]; again, no other *czc* genes were annotated on this plasmid. ACR3 is a very widespread transmembrane arsenic transporter [63]. We did not find evidence of regulators for the *ars* operon, or any other components of the operon, on this plasmid; however, it is not uncommon for elements of this pathway to be scattered throughout the chromosome [64]. Given the relatedness of the predicted functions, as well as their arrangement on the plasmid, it is possible that these genes in fact form a cassette related to metal resistance. One or more of these genes were found in 108 metagenomes, but only three metagenomes (including the metagenome corresponding to the sample from which this isolate was cultured) were found to contain all three.

### Differences between resistance genotype and phenotype of the *S*. *equorum* dust isolate

The reconstructed genome of the *S*. *equorum* isolate included 95 genes related to antibiotic resistance (**S4 Fig**) with two additional ARGs detected on its plasmids (both *lnuA* variants, **Fig 5**). Antibiotic resistance genes detected in the genome were part of the following antibiotic classes: multidrug resistance, penicillins (including methicillin), beta-lactams, fluoroquinolones, macrolides, aminoglycosides, tetracyclines, bacteriocins, glycopeptides, and others such as genes encoding trimethoprim resistance (see BioProject ID PRJNA554361).

Intriguingly, however, when this isolate was tested for resistance to clindamycin (lincosamide class), streptomycin (aminoglycoside), ampicillin (penicillins), chloramphenicol (chloramphenicol class), kanamycin (aminoglycoside), tetracycline (tetracyclines) and ciprofloxacin (fluoroquinolones), no resistance was observed (**Table 2**). The discrepancy between resistance genotype and phenotype has already been observed in other isolates [65] and can be attributed to several factors, such as the medium, the conditions in which the bacteria were grown, and regulation of the relevant genes and their translated proteins. For example, growth in Mueller Hinton broth may not be optimal to stimulate a resistance phenotype due to the absence of stress or other microbial competitors [66]. Thus overall, while our metagenomic survey indicates a sparse but quantifiable landscape of ARG in built environment dust, the mobility, functionality, and even viability of these ARG and their hosts may be limited under typical circumstances, thus fortunately decreasing their potential negative impact on human health.

## Discussion

This study was the first to combine metagenomic sequencing with cultivation-based approaches to identify mobilizable ARGs in indoor dust. We identified several MGEs and potentially mobile ARGs from built environment metagenomes, representing both the diversity of potential resistance mechanisms to which building occupants could be exposed and the diversity of host microbes carrying those elements. Of potential microbial hosts in these environments, we focused on *Staphylococcus*, resulting in a parallel phenotypic characterization, genome, and plasmid sequencing in cultivable isolates that carry MGEs and potentially mobile ARGs. The combination of these methods captured complementary aspects of the indoor resistome, demonstrating the diversity of this area remaining to explore.

Merely identifying a potential resistance gene sequence or mobile element in the environment does not indicate its functionality, let alone a public health risk. It is well known that not all genes that are annotated as ARGs in fact confer resistance, or vice versa [37]. For example, we identified *gidB*, which is associated with streptomycin resistance according to CARD; however, the isolate carrying this gene did not express the streptomycin resistance phenotype according to MIC tests (**Table 2**). In contrast, this isolate was observed to be highly resistant to ciprofloxacin (128 mg/L), which is in agreement with the presence of multiple genes with multiple copies associated with fluoroquinolone resistance, such as *qacA*, *emrB* or *norA* genes. Thus, as previously described from e.g. the gut [67], these observations demonstrate that there can be a discrepancy between the detection of antibiotic resistance genes in a built environment and the existence of a specific antibiotic resistance phenotype. Antibiotic resistance gene expression is likely also controlled by environmental stimuli, including antibiotics but also other substances, and the medium in which an isolate grows. It is also possible that antibiotic resistance gene was mis-annotated or that the annotation was based on the function of the gene in another species and that the function is not conserved in all species.

In parallel to metagenomics for hypothesis generation, we screened the culture isolates collected from the same samples to confirm the presence of ARG and MGE using whole genome sequencing and to test for the associated phenotypes. The original culture effort resulted in over 7,500 colonies. To narrow our screening efforts, we focused on culture-based characterization of staphylococci for several reasons. It is one of relatively few clades near-universally prevalent on human skin as a commensal, and as a result is widely distributed in the built environment; it can also drive significant opportunistic infection [68]. In fact, the clade represents a serious human health risk via antimicrobial resistance [59] in the built environment, often associated with high-touch surfaces where it can remain viable for days [69–71]. Finally, because one of our metagenomes contained a high relative abundance of *Staphylococcus*-associated reads, we were confident that we would find *Staphylococcus* within the culture isolates generated from that sample. This work supplements other studies of *Staphylococcus* as a commensal and opportunistic microorganism in the built environment. For example, DNA sequencing and comparative genomics coupled with phenotypic resistance testing was used to examine the relationship between nosocomial and commensal *S*. *epidermidis* isolates [72]. In another study, metagenomic sequencing coupled with *in vivo* testing was used to assess the contribution of *S*. *epidermidis* and *S*. *aureus* strain-level variation to atopic dermatitis [73].

The combination of sequencing and culture data resulted in two key observations. First, the aspects of antimicrobial resistance and gene mobility characterizable by the sequencing and culture-based methods were highly complementary. Even in a small number of examples, not all potential resistance elements in the sequenced dataset were functional under the conditions evaluated, nor all mobilization mechanisms. Second, the strain-specific diversity of intrinsic and acquired ARG is extremely important in linking an annotated ARG to an antibiotic resistance phenotype. This has been widely observed in other sources, and this study suggests the potential health relevance of mobilizable ARGs in this new, increasingly relevant setting.

The detected MGEs were in proportion mainly transposons, followed by integrons and plasmids, probably reflecting the apparent overrepresentation of transposases over integrases and plasmid-associated genes in the NCBI nr database [74–76]. Nevertheless, potentially mobile multidrug efflux pump genes were located indiscriminately on transposons, integrons and plasmids.

In addition to multidrug efflux pump genes, potentially mobile aminoglycoside resistance and tetracycline genes were frequently detected; these genes are also among the most abundant and frequent ARGs in all dust samples. The high ubiquity and abundance of aminoglycoside and tetracycline resistance genes in dust samples might be related to their location on transposons and integrons. No genes of these classes of antibiotics were found on plasmids in the dust metagenomes, which can be explained by a rare presence of these genes on plasmids or more likely a low representation and detection of plasmid sequences in metagenomes, as detection of plasmids and associated gene in metagenomes is often challenging.

We investigated a specific dust isolate of clinical relevance (*S*. *equorum*), which contained several of both intrinsic and the plasmidic ARGs. While comprehensive studies of antibiotic resistance in *S*. *equorum* are not available, the presence of multiple ARG is well documented in other members of the *Staphylococcus* genus. For example, pangenomic analysis of *S*. *epidermidis* showed that this species contains 28 different resistance gene types, conferring resistance to 31 different antibiotics, and nearly all isolates of this species have at least one ARG and at most 9 [77]. Of *S*. *epidermidis* isolates collected from medical devices, 75% were methicillin-resistant, while only 28% of commensal isolates displayed this phenotype [72]. Over half of medical device-associated isolates were resistant to trimethoprim and clindamycin (lincosamide) [72]. Our isolate demonstrated a much higher number of intrinsic ARGs (*i*.*e*. located on the genome of *S*. *equorum*) than acquired ARGs (*i*.*e*. located on the four reconstructed plasmids). Only one plasmid of this isolate harbored ARGs, and the two ARGs were both annotated as *lnuA* genes encoding resistance to lincosamides (**Fig 5**). The resistance phenotype of this isolate further suggested these two genes are expressed in the tested conditions (no other gene related to lincosamide resistance was detected on the genome), while a number of intrinsic ARGs were not reflected in the expressed resistance phenotype (**Table 2**). Unsuccessful conjugation and transformation assays (**Supporting Information**) with the plasmid carrying the *lnuA* genes with a *S*. *aureus* ATCC 25923 strain suggested that the plasmid might not be transferable, although transfer may occur in other contexts or with other hosts.

This study demonstrated that dust contains on average a lower total abundance of MGE than drinking water or cattle stool, while harboring a significantly higher total ARG abundance than drinking water and lower than cattle stool (**Fig 3**). Cattle stool environment is particularly loaded in ARG due to the use of antibiotics in cattle breeding and may be a pathway mobile ARGs can be shared between animals and humans [2]. Drinking water is increasingly monitored for the presence of ARGs, which are regarded as emerging environmental pollutants [4]. These results thus raise the idea that although dust shows theoretically a lower potential to transfer genes than drinking water, it constitutes a substantial reservoir of ARGs.

In this study, we found evidence that at least one identified mobile ARG, the *lnuA* gene, located on a plasmid of an environmental strain according to our dust metagenomes, is present in viable dust isolates. This gene has been detected in multiple copies in two samples in metagenomes. We confirmed through a cultivation-based approach that the *lnuA* gene is actually present on plasmids as predicted from the dust metagenomes. This combination of metagenomics and cultivation brings new insight into the actual risk of ARG dissemination and highlights the actual importance of assessing when possible the transferability of detected ARGs [49].

High-throughput metagenomic sequencing gives a broad overview of the taxonomic diversity and functional genes present in a given sample. However, this approach has several limitations. For samples where library concentrations have been normalized, the number of reads produced per sample is even most of the time; however, some differences in the number of reads produced per sample still persist and can be important in some cases. In addition, environmental samples rarely show the same level of diversity, and samples with lower diversity demonstrate a better coverage depth than those with higher diversity for a similar number of reads produced. The two dust datasets used in this study come from two different runs, with a different number of samples included in each run (122 samples over 2 Illumina HiSeq runs 2x150bp for one dataset versus 44 samples over one Illumina MiSeq run 2x150bp for the other) and show differences in coverage depth.

In order to limit the effect of coverage differences, we normalized the abundance of mobile ARGs, which we assessed through the calculation of their coverage, based on the total number of reads in the corresponding sample. Sequencing depth and diversity in a sample influence coverage, and the coverage impacts the number and the length of contigs obtained through assembly. Coverage should thus influence the total number of MGE and mobile ARGs detected in each sample, and one would expect to find more MGEs and associated potentially mobile ARGs in samples with higher coverage.

This work is one of the first studies testing actual possible transfer of ARGs identified priorly through a metagenomic approach. Our results suggest that mobile ARG detected using metagenomics but not confirmed through cultivation and molecular biology approach, cannot constitute systematically a health risk and that further investigation with different complementary approaches, namely cultivation and molecular biology are needed to assess the actual real risk associated to the detection of ARG and mobile ARG in a public environment. This hybrid approach is important for interpreting data related to ARGs in environments as heterogeneous as dust.

An investigation of dust isolates at a much larger scale is needed to provide an accurate representation of the real potential for dissemination of ARGs in dust microbial communities. Further study is needed to specifically examine the conditions necessary for ARG expression and MGE transfer. Gene annotation is an imperfect process. As such, expression and function of putative ARGs should be confirmed by measuring transcripts and corresponding phenotypes. Plasmid transfer may require very specific conditions to happen or may not even happen. Plasmid host ranges can vary significantly according to the plasmid and to the available hosts, some plasmids having broad host range and other narrow host range [78]. In addition, plasmids show variable size and copy number which can influence their dissemination. Finally, the expression of plasmid located genes is also dependent on the compatibility between the host and the plasmid [39]. Integration of complementary approaches such as molecular biology tools (e.g. RT-PCR on ARGs; transcriptomics) and cultivation (conjugation and transformation assays) will provide a more accurate picture of the actual risk of ARG detection for human health.

### Conclusion

In summary, this study shows that dust within modern buildings is a reservoir of ARGs and a possible vector for bidirectional transfer of ARGs between the human microbiome and the outdoor environment. Based on these findings and on the developed hybrid approach in this study, we should consider the impact of building design and operation (including selection of cleaning products, building construction materials, home furniture and personal daily care products) on indoor dust microbial communities and the spread of ARGs, in addition of their potential direct and indirect effects on human health. Finally, and most importantly, in order to provide the most accurate risk assessment related to ARGs for human health, future studies should attempt to include, as much as possible and when possible, tests for actual transferability of mobile ARGs identified through metagenomic approaches.

## Supporting information

S1 Fig Pie chart of the proportions of the different types of mobile genetic elements investigated in this study. (TIFF)

S2 Fig Estimated average coverage for each dust metagenome computed with Nonpareil [50]. (TIFF)

S3 Fig Halla plot of Pearson correlations between ARG abundance, and bacterial and viral genus abundance in collected dust samples. (TIFF)

S4 Fig Genome of the *S*. *equorum* dust isolate reconstructed from whole genome sequencing (Illumina technology). The genome was reconstructed using SPades and represented using DNAPlotter. Red dashes on the second circle indicate annotated antibiotic resistance genes on the forward DNA strand while the third circle shows the ones detected on the reverse strand; orange dashes on the first circle: other annotated genes, light grey: repeat regions, long turquoise dashes: mobile genetic element genes, dark green and dark purple: GC plot, light green and purple: GC skew.
(TIF)

S1 Text Additional methods. (DOCX)
