# Supplementary figures and images for: Mobilizable antibiotic resistance genes are present in dust microbial communities
Source: PLoS Pathog. 2020 Jan 23;16(1):e1008211. doi: 10.1371/journal.ppat.1008211 (PMC6977718; doi:10.1371/journal.ppat.1008211)

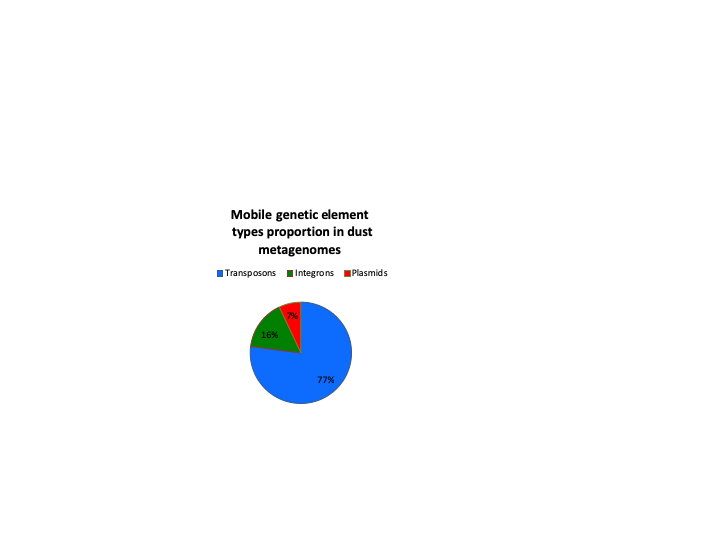

Supplement: S1 Fig — (TIFF) [file ppat.1008211.s001.tiff]

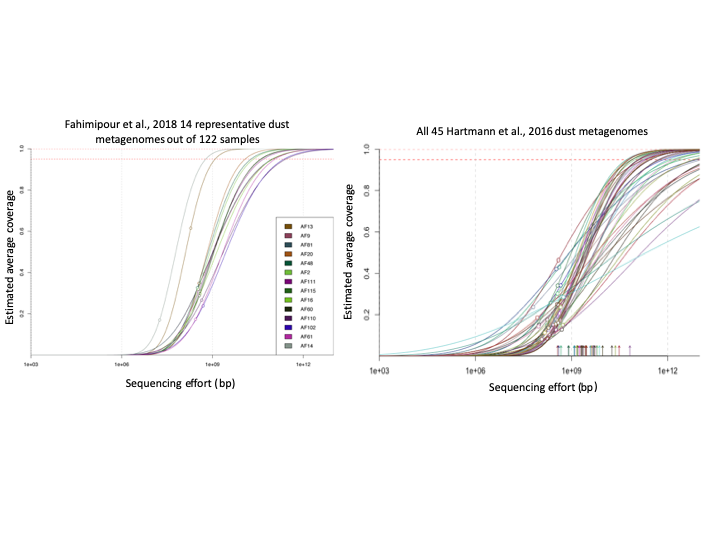

Supplement: S2 Fig — (TIFF) [file ppat.1008211.s002.tiff]

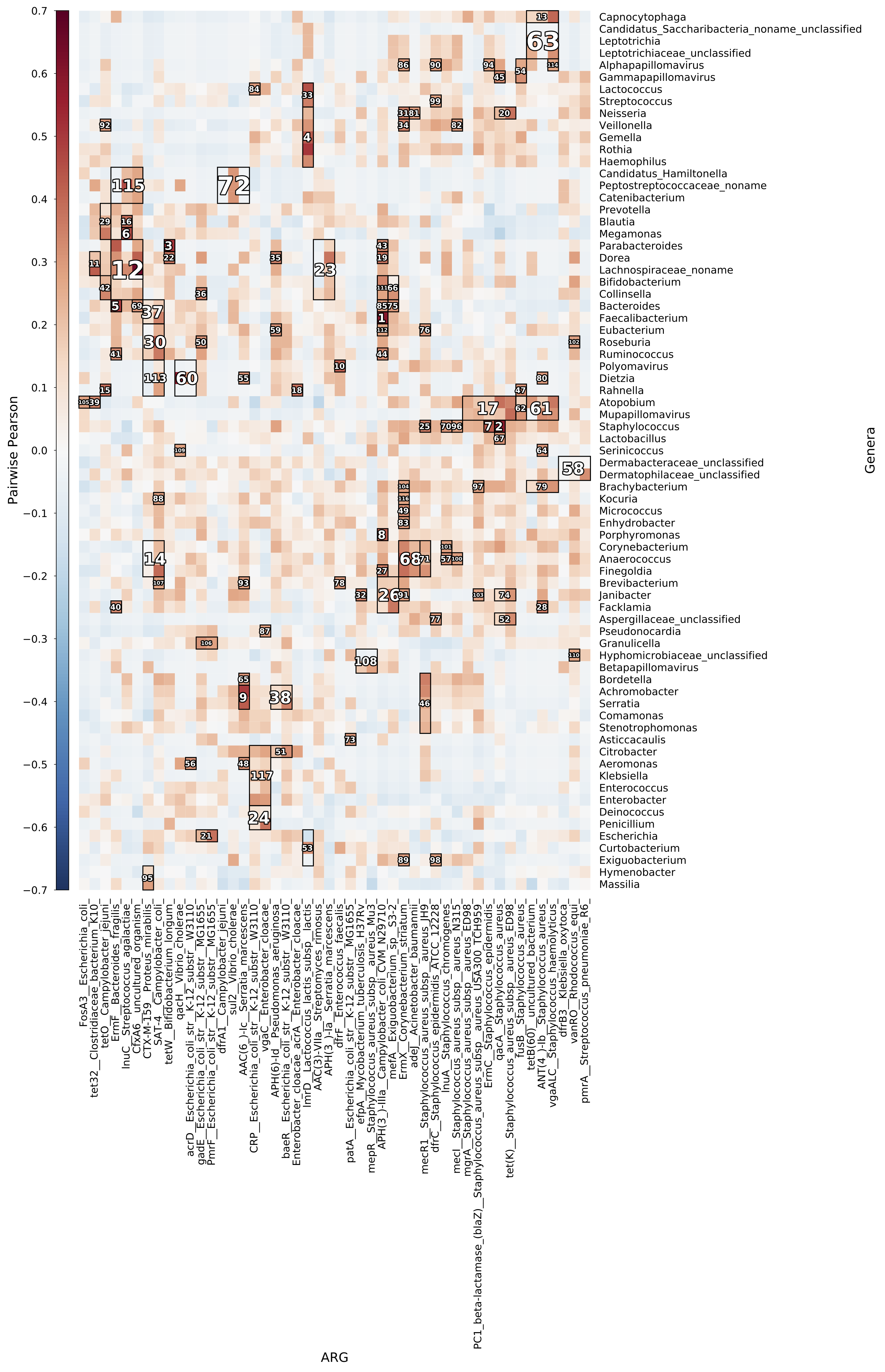

Supplement: S3 Fig — (TIFF) [file ppat.1008211.s003.tiff]

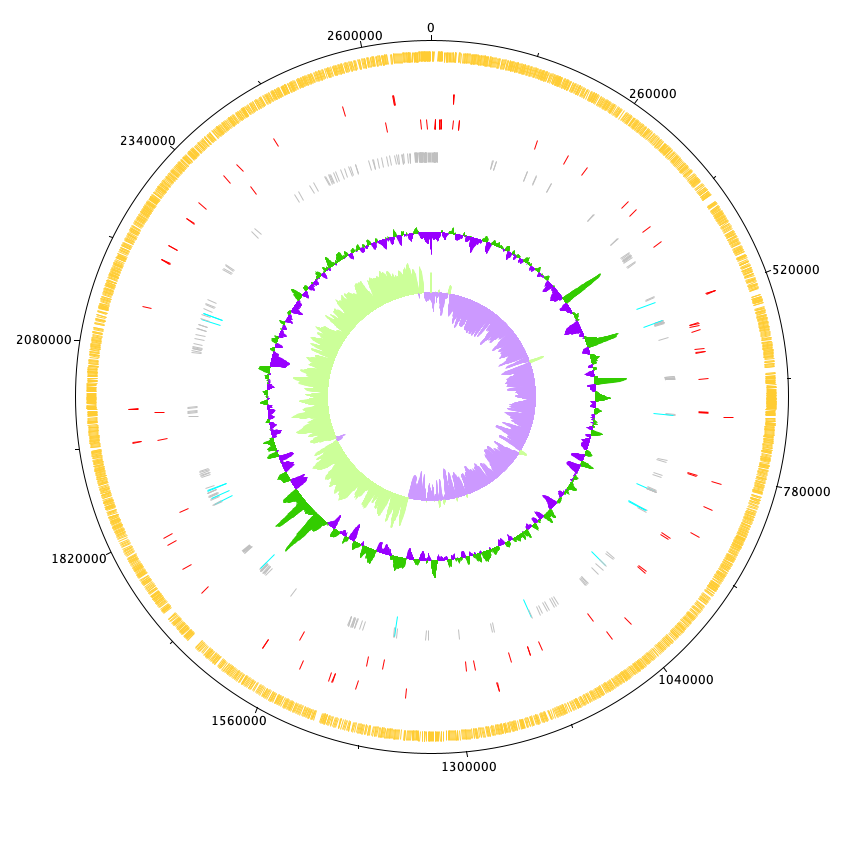

Supplement: S4 Fig — The genome was reconstructed using SPades and represented using DNAPlotter. Red dashes on the second circle indicate annotated antibiotic resistance genes on the forward DNA strand while the third circle shows the ones detected on the reverse strand; orange dashes on the first circle: other annotated genes, light grey: repeat regions, long turquoise dashes: mobile genetic element genes, dark green and dark purple: GC plot, light green and purple: GC skew. (TIF) [file ppat.1008211.s004.tif]
