# Supplementary material for: Mobilizable antibiotic resistance genes are present in dust microbial communities
Source: PLoS Pathog. 2020 Jan 23;16(1):e1008211. doi: 10.1371/journal.ppat.1008211 (PMC6977718; doi:10.1371/journal.ppat.1008211)
Supplement: S1 Text — (DOCX) [file ppat.1008211.s005.docx]

**Sample collection, DNA extraction and metagenomic sequencing**

Dust was collected as described in Ashkann et al., 2018. Briefly dust was collected at each site using a vacuum fitted with Dustream collectors (Indoor Biotechnologies, Charlottesville, VA, USA). Collector filters (40 micron mesh) containing the vacuumed dust were placed into sterile Nasco Whirl-Paks bags (Nasco, Fort Atkinson, WI), and stored in dark conditions at room temperature during collections. Collection took place from 2016 July 18 to 2016 November 21. Each sample was then homogenized and 0.25 g of dust was aliquoted into sterile 2 mL tubes and stored at -80 °C until DNA extraction.

The DNA from dust aliquots was extracted using the MoBio PowerLyzer PowerSoil DNA Isolation Kit (MoBio, Carlsbad, CA, USA) protocol. To perform a metagenomic analysis, 1 ng of gDNA from each sample was prepared using the Illumina Nextera XT DNA Library Prep Kit, along with the corresponding Illumina Index Kits v2 set A and set B, following the manufacturer’s instructions through the amplification step. Amplified products were purified with a modified bead-based DNA clean-up protocol using Mag-Bind RxnPure Plus by Omega Bio-tek, quantified using Quant-iT dsDNA assay kit, and pooled with equal concentrations of product using an Eppendorf epMotion 5075 robot. Libraries were sequenced on an Illumina HiSeq 4000 150bp paired end reads (insert size ranged from 250-1000bp).

**Conjugation assay with dust isolates and designed primers to track donor and recipient**

Based on dust isolate taxonomy and plasmid sequences, the *S. equorum* dust isolate was chosen as a donor (carrying a *lnuA* gene on a plasmid) to test for plasmid transfer with *S. aureus* ATCC 25923 lab strain as the recipient. The MIC of each donor and recipient was assessed (described above). The donor was selected for its resistance to 0.5 mg/L clindamycin, and the recipient for resistance to 4 mg/L streptomycin; transconjugants should be resistant to both clindamycin and streptomycin resistance at 0.5 mg/L and 4 mg/L, respectively. The conjugation assay between each donor and recipient was performed as described in Wang et al., 2018 by mating equal density of donor and recipient (approximately 10^8^ cells/mL) in triplicate in liquid culture. The conjugation and/or transfer of the plasmid was also tested by depositing a drop of 10µL of donor culture (in TSA) in exponential phase on a TSA plate supplemented with clindamycin and streptomycin (0.5 and 4 mg/L, respectively) on top of a 10µL drop of recipient culture (in TSA) in exponential phase. The plate was then placed in the incubator at 37°C for 24h.

In order to track the transfer of the plasmidic *lnuA* genes in potential transformants or transconjugants, PCR assays were performed using primers that were designed to specifically amplify plasmidic *lnuA* genes detected in the “unitig 10” plasmid of the *S.equorum* dust isolate. The lnuA primers are the following: lnuAF 5’-AGCTCAGCCACGTCC-3’ (Tm=55.6°C); lnuAR 5’-ATACGCTAAGCCACGTCC-3’ (Tm=54.6°C). In order to track recipients and potential transformants or transconjugants, new specific primers targeting the thermocnuclease of *S. aureus* ATCC 25923 were designed: nucAF 5’-AGCTCAGCAAATGCATCACAA-3’ (Tm=55.1°C); nucAR 5’-ATACGCTAAGCCACGTCCAT-3’ (Tm=55.3°C). The run PCR program was the following: one initial step at 95 °C for 2 min, a second step at 95 °C for 15 s, a third step at 55 °C for both lnuA and nucA primer set for 60 s, a fourth step with heating to 72 °C for 45 s, a fifth step with cooling to 72 °C for 5 min. The second, third and fourth steps were repeated for 30 cycles on an Eppendorf MasterCycler Nexus Thermal Cycler. All primer sets were tested for specificity by cross-checking with PCR on both donor and recipient.
